# Spontaneous transformation of human granulosa cell tumours into an aggressive phenotype: a metastasis model cell line

**DOI:** 10.1186/1471-2407-8-319

**Published:** 2008-11-04

**Authors:** Misa Imai, Miho Muraki, Kiyoshi Takamatsu, Hidekazu Saito, Motoharu Seiki, Yuji Takahashi

**Affiliations:** 1 Division of Reproductive Medicine, Department of Perinatal Medicine and Maternal Care, National Center for Child Health and Development, Tokyo 157-8535, Japan; 2 Division of Cancer Cell Research, Institute of Medical Science, the University of Tokyo, Tokyo 108-8639, Japan; 3 Department of Obstetrics and Gynecology, Tokyo Dental College Ichikawa General Hospital, Chiba 272-8513, Japan

## Abstract

**Background:**

Granulosa cell tumours (GCTs) are frequently seen in menopausal women and are relatively indolent. Although the physiological properties of normal granulosa cells have been studied extensively, little is known about the molecular mechanism of GCT progression. Here, we characterise the unique behavioural properties of a granulosa tumour cell line, KGN cells, for the molecular analysis of GCT progression.

**Methods:**

Population doubling was carried out to examine the proliferation capacity of KGN cells. Moreover, the invasive capacity of these cells was determined using the *in vitro *invasion assay. The expression level of tumour markers in KGN cells at different passages was then determined by Western blot analysis. Finally, the growth and metastasis of KGN cells injected subcutaneously (*s.c.*) into nude mice was observed 3 months after injection.

**Results:**

During *in vitro *culture, the advanced passage KGN cells grew 2-fold faster than the early passage cells, as determined by the population doubling assay. Moreover, we found that the advanced passage cells were 2-fold more invasive than the early passage cells. The expression pattern of tumour markers, such as p53, osteopontin, BAX and BAG-1, supported the notion that with passage, KGN cells became more aggressive. Strikingly, KGN cells at both early and advanced passages metastasized to the bowel when injected *s.c. *into nude mice. In addition, more tumour nodules were formed when the advanced passage cells were implanted.

**Conclusion:**

KGN cells cultured *in vitro *acquire an aggressive phenotype, which was confirmed by the analysis of cellular activities and the expression of biomarkers. Interestingly, KGN cells injected *s.c. *are metastatic with nodule formation occurring mostly in the bowel. Thus, this cell line is a good model for analysing GCT progression and the mechanism of metastasis *in vivo*.

## Background

GCTs are a relatively uncommon neoplasm; the incidence of GCTs ranges from 1.6–3.0% in all cases of ovarian tumour and comprises about 10% of all cases of ovarian cancer [1]. They belong to the sex-cord stromal tumours [2], and are classified as juvenile or adult, although the majority of GCTs occur in menopausal women [3]. GCTs are known to retain numerous characteristics of native granulosa cells, such as the expression of active FSH receptor, inhibin, and estrogen [4-6]. Although GCTs have a malignant potential, they are often indolent and have a propensity for late recurrence [7,8]. Up to 53% of all cases lead to metastases within 5 years and studies encompassing long-term follow-up have shown high mortality rates, with about 50% of women dying from the disease within 20 years of diagnosis [9,10]. Although there have been extensive studies on the biology of normal granulosa cells [11,12], much knowledge of the molecular mechanism by which transition from promotion to the progression stage occurs in GCTs remains unknown.

To date, only seven human granulosa cell lines have been established, [1,13-18] although several animal granulosa cell-derived cell lines have been reported [14]. Of these, KGN cells were generated from a GCT that recurred in the pelvic region, and were shown to have detectable aromatase activity [1]. KGN cells have an abnormal karyotype (45, XX, 7q-, -22) which is probably related to the tumourigenic character of this granulosa cell, as frequent abnormalities in chromosome 7 have been reported in ovarian tumours [19,20]. Interestingly, KGN cells revealed a unique characteristic and grew progressively faster during passages in our preliminary experiment.

Here, we investigated the specific characteristics of KGN cells towards understanding the molecular pathogenesis of GCTs. Because KGN cells grew much faster after passages in culture, we investigated their cellular characteristics, such as proliferation capacity and invasiveness, during passages *in vitro*. We then investigated the behaviour of these cells *in vivo *with the use of subcutaneous xenografts at different passages.

## Methods

### Reagents

Hoechst 33342 and Mitomycin C from *Streptomyces Caespitosus*, luteinizing hormone (LH) and follicle stimulating hormone (FSH) were purchased from Sigma Chemical Co. (St. Louis, MO, USA). Mouse monoclonal antibody against p53 and osteopontin and rabbit polyclonal antibodies against BAG-1 and BAX were purchased from Santa Cruz Biotechnology Inc. (Santa Cruz, CA, USA). Goat anti-mouse IgG-HRP and goat anti-rabbit IgG-HRP were purchased from Pierce Chemical Co. Ltd. (Woburn, MA, USA) and Chemicon International Co. Ltd. (Temecula, CA, USA), respectively.

### Cell Culture

KGN cells were obtained from RIKEN Bioresource Center (Tsukuba, Japan). Early (fewer than P10) and advanced (more than P47) passage KGN cells were maintained as described previously [21]. For detailed characterisation, the cells were cultured in a medium supplemented with 10% charcoal/dextran-treated FBS (delipidated FBS, Thermo Fishers Scientific Inc., South Logan, UT, USA), 0.1 IU/ml LH or 1IU/ml FSH. Cells were routinely passaged after brief exposure to 0.25% trypsin with 0.02% EDTA in PBS (-).

### Evaluation of cellular morphology, proliferation and invasion

The morphology of KGN cells was observed under a phase-contrast microscope (IX-71; Olympus, Tokyo, Japan). The cellular proliferation was measured by population doubling of KGN cells in a logarithmic growth phase at a starting concentration of 2 × 10^5 ^cells/dish in 60 mm Petri dishes (Falcon, BD Japan, Tokyo, Japan). The number of KGN cells was determined at 24 h interval for 4 days while changing the medium every other day. To determine the proliferation rate of KGN cells at Day 4, a colorimetric TetraColor ONE ELISA kit (SEIKAGAKU CORPORATION, Tokyo, Japan) was utilised. Cells were initially seeded at a density of 1 × 10^4 ^cells/well in 96-well plates and the subsequent assay was carried out according to the manufacturer's instructions. For invasion assay, KGN cells (1 × 10^5 ^cells/well) suspended in serum-free medium were seeded on the upper chamber coated with Matrigel (200 μg/ml, Becton Dickinson, San Jose, CA, USA). FBS (10%) was added to the medium in the lower chamber and incubated for 12 h to allow cell invasion, and then cells were fixed with 100% methanol. To exclude the possibility that invasiveness was overestimated by distinct proliferation capacity of different passage cells, they were treated with 10 μg/ml Mitomycin C for 2 h at 37°C in a CO_2 _incubator and subjected to the invasion assay. The cells retained in the upper chamber were scraped off and stained with 10 μM Hoechst 33342 for 30 min at room temperature. The number of cells that invaded through the chamber was counted using an epi-fluorescence microscope. The experiments described above were triplicated.

### Chromosome analysis

The chromosomes were examined in exponentially growing early and advanced passage KGN cells (early: P6; advanced: P53) in an *in vitro *culture. The karyotype of 10 KGN cells was analysed by standard trypsin G-banding, as described previously [1].

### Western blot analysis

Cells were lysed in RIPA buffer containing protease inhibitor cocktails I and II (1:100 dilution, Sigma Chemical Co) for 2 h at 4°C, and the insoluble materials were removed by centrifugation (15,000 rpm, 15 min). The protein concentration of samples was determined using a micro BCA assay kit (Pierce Chemical Co. Ltd., Rockford, IL, USA). Ten or fifty micrograms of samples per lane were separated on a 12.5% reducing SDS polyacrylamide gel, and transferred onto a PVDF membrane (Immobilon-P, Millipore Japan, Tokyo, Japan). After blocking with 10% goat serum plus 90% Blockace (SnowBrand, Tokyo, Japan) at room temperature for 1 h, the membranes were treated with the primary antibody and subsequently with the secondary antibody. SuperSignal West Femto Maximum Sensitivity Substrate (Pierce, Rockford, IL, USA) was used for visualisation, and the signal was developed on an X-ray film (Amersham Bioscience, Piscataway, NJ). The band intensity of each protein was measured using ADOBE Photoshop Element 3.0 software and the background was subtracted. It was then divided by the band intensity of β-actin for normalisation.

### Tumour xenografts

The experiments were approved by the Animal Ethics Committee at the National Center for Child Health and Development, Japan. Six-week-old female BALB/c *Foxn1*/*Foxn1 *mice (nude mice) were purchased from Sankyo Labo Service Co. Ltd. (Tokyo, Japan). The early or advanced passage KGN cells (5 × 10^6 ^cells) were harvested, resuspended in 200 μl of PBS, and injected *s.c. *into both flanks of each mouse (early; n = 9, advanced; n = 9). Mice were euthanized and examined for tumour generation after 3 months, and the number of nodules formed in the bowel was counted. The experiments were quadruplicated.

### Histological evaluation

For light microscopic analysis, the metastases formed in the bowel were fixed with 4% formaldehyde, paraffin-embedded, sectioned at 4 μm intervals and stained with hematoxylin-eosin.

### Semi-quantitative RT-PCR analysis

The small intestine with or without metastases was obtained from four mice in each group (control, the 'early', and the 'advanced' groups) and total RNA was isolated with the use of ISOGEN RNA isolation reagent (Nippon Gene Co. Ltd., Tokyo, Japan). Reverse transcription was conducted, as described previously [21]. The expression of p53 was monitored by PCR amplification. The specific primers for human p53 and GAPDH and cycles used for each gene were as follows; p53 forward primer: CAGCCAAGTCTGTGACTTGCACGTAC, p53 reverse primer: CTATGTCGAAAAGTGTTTCTGTCATC, 35 cycles; GAPDH forward primer: ACCACAGTCCATGCCATCAC, GAPDH reserve primer: TCCACCACCCTGTTGCTGTA, 28 cycles. The conditions for PCR amplification were: initial denaturation at 94°C for 5 min, followed by the indicated cycles of denaturation at 94°C for 45 s, annealing at 57°C for 45 s, and elongation at 72°C for 1 min, with a final extension of 72°C for 15 min. The image analysis of the PCR product was performed as described previously [21].

### Statistical analysis

The average data on population doubling, proliferation, invasion and metastasis were presented as means with SEM from three independent experiments. Statistical analyses were conducted by Student's or modified *t*-test using Microsoft Excel software. Differences were considered statistically significant when P < 0.05.

## Results

### Characterisation of KGN cells in vitro

**Figure 1:**
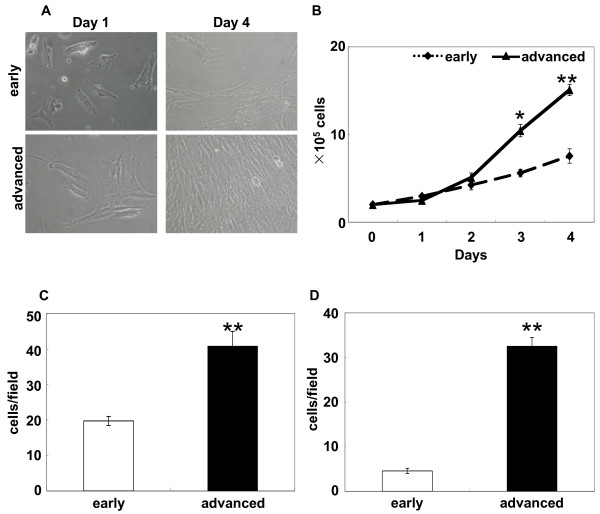
**Characterization of KGN cells at different passages.** (A) Morphology of KGN cells at Days 1 and 4 (P9 vs. P58). (B) Population doubling measured for 4 days using the early and advanced passage KGN cells seeded at 2 × 10^5 ^cells/dish (P9 vs. P58). (C, D) Invasiveness of different passage KGN cells (P6 vs. P53). Cells in serum-free medium at a concentration of 1 × 10^5 ^cells/well were subjected to an invasion assay using transwell chambers coated with Matrigel for 12 h (C; normal cells, D; Mitomycin C-treated cells). The proliferation of Mitomycin C-treated cells was blocked, as determined by the TetraColor One proliferation assay. All experiments were triplicated and the data are shown as the mean number with SEM. Statistical difference was analysed by Student's *t*-test (*P < 0.05).

KGN cells appeared to grow faster during passages in our preliminary experiments. For detailed characterisation of KGN cells, early and advanced passage cells were cultured, and the proliferation capacity was monitored. Growth of the advanced passage cells turned out to be significantly faster than that of the early passage cells, determined by population doubling evaluation (Fig 1A and 1B, about 2-fold increase at Days 3 and 4, P < 0.05, P < 0.01). The behavioural changes of KGN cells at different passages occurred without any morphological changes at Days 1 and 4 (Fig 1A).

Next, we conducted an *in vitro *invasion assay to assess the characteristic features of the tumours. Along with increased proliferative activity, the advanced passage cells were 2-fold more invasive than the early passage cells (Fig 1C, P < 0.01). Since the advanced passage cells were more proliferative than the early passage cells, KGN cells were treated with Mitomycin C to prevent proliferation during the invasion assay. Loss of proliferative capacity of the advanced passage cells was confirmed by the TetraColor ONE proliferation assay (OD450; untreated early passage cells: 0.10; untreated advanced: 0.18; treated early: 0.09; treated advanced 0.10). Under the conditions of the assay, the advanced passage cells remained more invasive than the early passage cells (Fig 1D, P < 0.01), suggesting that the increased invasiveness was not related to proliferation.

**Figure 2:**
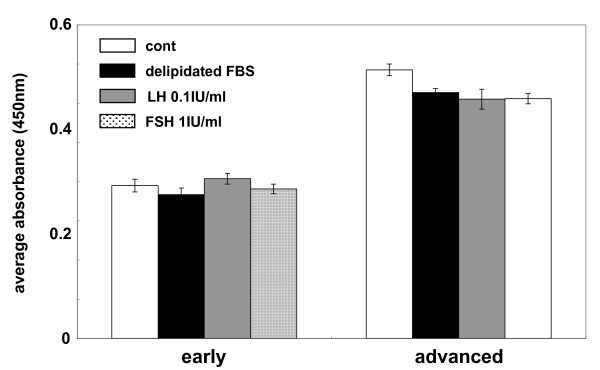
**Proliferation of KGN cells at different passages.** The early (P6) and advanced (P58) passage KGN cells were cultured in 10% delipidated FBS, 0.1 IU/ml LH, or 1 IU/ml FSH, seeded at 1 × 10^4 ^cells/well in 96-well plates and subjected to the proliferation assay. The experiments were triplicated and the data are shown as the mean absorbance with SEM (at OD 450 nm). Statistical analysis was conducted using Student's *t*-test.

Because serum factors and hormones may be involved in GCT progression, KGN cells were cultured in a medium containing delipidated FBS, LH or FSH, and subjected to the proliferation assay. As shown in Fig 2, none of these treatments affected the characteristics of the early and advanced passage cells.

### Karyotype analysis

G-banded karyotype analyses of 10 early and advanced KGN cells revealed that all the cells exhibited 45 chromosomes (data not shown), as described previously [1].

### Expression of various tumour markers in KGN cells at different passages

**Figure 3:**
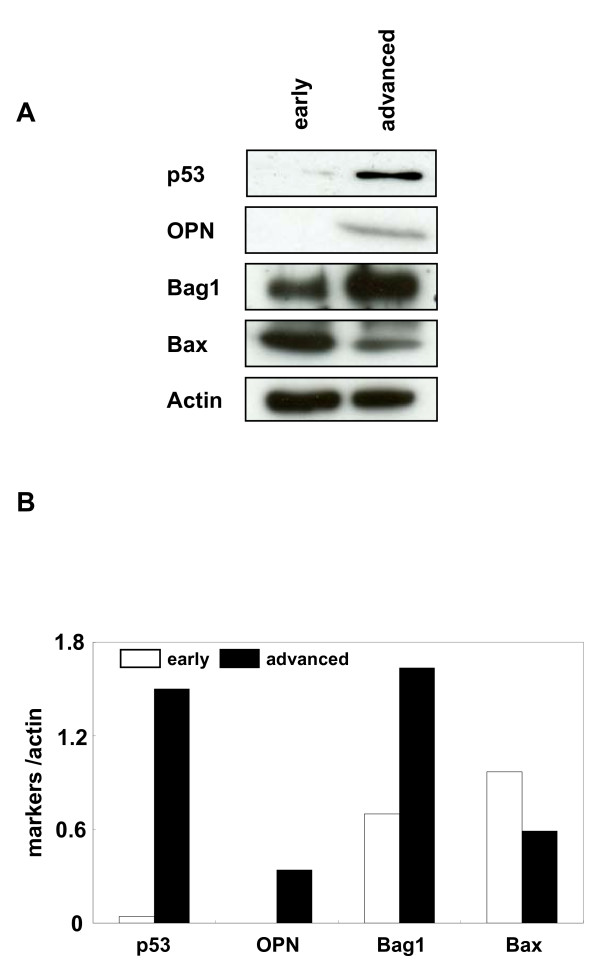
**Expression of tumour markers in KGN at different passages.** Western blot analysis of KGN cells at P7 and P51 was conducted to examine the expression level of p53, osteopontin, BAX and BAG-1. Ten μg (for BAX and BAG-1) or 50 μg (for p53 and osteopontin) of whole cell extract were separated on a 12.5% reducing gel. The lower panel represents the mean intensity of each band normalised by dividing with the intensity of β-actin.

Subsequently, we examined the expression level of malignant tumour markers in the early and advanced passage cells (Fig 3A, B). Initially, we determined the expression level of p53, which has been reported to be a prognostic marker of metastasis in granulosa cell tumours [9,22]. As expected, the expression level of p53 was up-regulated in the advanced passage cells. Next, we performed immunoblots for osteopontin, which is a biomarker of tumour progression [23], and found that it markedly increased in the advanced passage cells. Furthermore, we examined the expression of BAX and BAG-1, which are known biomarkers of apoptosis [24]. As expected, BAX was up-regulated, whereas BAG-1 was down-regulated in the advanced passage cells, suggesting that the advanced passage cells might be resistant to apoptosis. These results strongly indicate that KGN cells became aggressive during passages *in vitro*.

### Tumour growth *in vivo*

**Figure 4:**
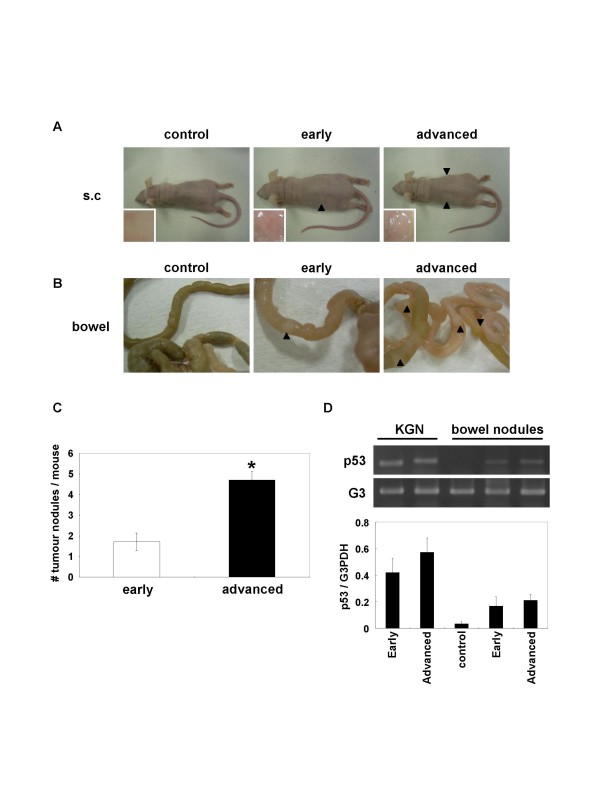
Growth and metastases of KGN cells in nude mice. KGN cells at the early (P7) and advanced (P58) passages were transplanted in the flanks of nude mice at a concentration of 5 × 10^6 ^cells. After 3 months, the growth of KGN cells at the injected region and metastases in the bowel were examined. (A) Growth of KGN cells (P7 and P59) at the injected regions shown by arrowheads. Tumour growth under the skin is shown in the enlarged windows. (B) Metastases of KGN cells in the bowel of nude mice. Arrowheads indicate metastases (nodules). The metastases were about 3 mm × 3 mm in size and were observed in 7 out of 9 mice. (C) The number of tumour nodules formed in the bowel was counted from 7 nude mice. Data are shown as the mean number of nodules with SEM. Statistical difference was analysed by Student's *t*-test (*P < 0.05). These experiments were quadruplicated. (D) Semi-quantitative RT-PCR analysis of human p53 in intestines with or without tumour nodules. Total RNA was isolated from the small intestine with or without nodules in each experimental group, and PCR analysis was performed using human-specific primers for p53. The upper panel represents a typical digital photograph taken on a transilluminator. Lower panel represents the averaged band intensity of p53 with SEM from four independent experiments.

Further characterisation of KGN cells was performed using an *in vivo *tumour growth assay in nude mice. As shown in Fig 4A, the xenograft of the advance passage cells developed faster than that of the early passage cells at the region where KGN cells were injected, and larger clumps under the skin were observed (in the small windows of Fig 4A). More interestingly, KGN cells at both the early and advanced passages metastasized from the subcutaneous transplanted region to the bowel, especially in the submucosa of the small intestine, but not to the other tissues (metastases in the bowel were seen in 7 out of 9 mice transplanted) (Fig 4B). Although an increased number of nodules were formed in the bowel by transplantation of the advanced passage cells (Fig 4C, P < 0.05), the size of nodules obtained using both passage cells was similar (3 mm × 3 mm).

**Figure 5:**
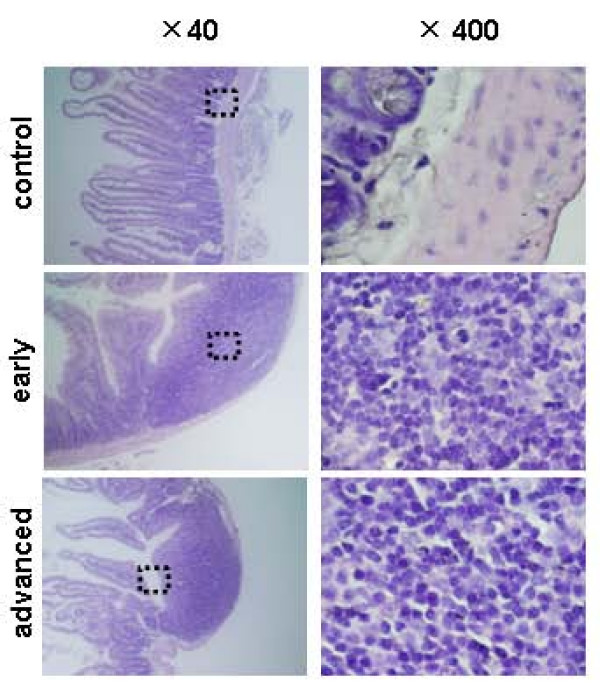
**Histology of metastases in nude mice.** The bowel containing the tumour nodules was paraffin-embedded, sectioned at 4 μm intervals and stained with hematoxylin-eosin. The left panels represent the lower magnification of the bowel with or without tumour nodules (×40, P7 vs. P59). The squared regions in the left panels are enlarged in the right panels (×400).

To confirm the origin of nodules, the expression of a GCT-specific marker in the nodules was examined by RT-PCR using human-specific primers. As shown in Fig 4D, human p53 mRNA was found to be expressed in the nodules, suggesting that the nodules originated from KGN cells. Moreover, histological analysis revealed that the cells at both passages exhibited a coffee bean-like nuclear appearance, which is typically observed in specimens of granulosa cell origin (Fig 5). These results demonstrated that KGN cells metastasized from the subcutaneous region to the bowel.

## Discussion

In the previous study, KGN cells were shown to retain properties of normal granulosa cells, such as aromatase activity and progesterone synthesis [1]. Despite the fact that KGN cells can be maintained in more than 100 passages, their native properties are unchanged during passages. Moreover, we confirmed progesterone synthesis in the advanced passage cells in response to cAMP, and that chromosomal instability did not occur during passages determined by G-band analysis (data not shown). Therefore, the transformation of KGN cells during passages occurs without loss of their native characteristics.

The mechanism responsible for the phenotypic transformation of the cells during passages remains uncertain. One possibility is that a minor population of aggressive GCTs included in the early passage cells prevailed during passages, resulting in an overall aggressive character. Although cloning of the early passage cells (7 clones examined) did not show any changes in phenotype among the clones (data not shown), this possibility could not be ruled out. The second possibility is that oxidative stress gained during *in vitro *culture may have prompted the transformation. Although administration of hydrogen peroxide to the culture of KGN cells did not affect their behaviour in our study (data not shown), it is likely that oxidative stress during *in vitro *culture may induce genome instability and mutation in these cells. The third possibility is that hormones, such as estrogen and progesterone, secreted from KGN cells influence the transformation. Although high expression level of estrogen receptor β (ERβ), a potent suppressor of proliferation, has been reported in GCTs [25], direct interaction between ERβ and NF-κB might be required for GCTs survival [26]. However, exogenous administration of hormones, such as estrogen, LH and FSH, to the culture did not enhance the transformation. Another possibility might be that cytokines and other growth factors present in FBS may influence the cellular phenotype, although we determined that at least EGF did not influence on the proliferation of KGN cells (data not shown). Therefore, further analysis is required to elucidate the exact mechanism involved in the transformation of KGN cells *in vitro*.

Spontaneous changes in cellular characteristics during *in vitro *culture are, more or less, common among different cell types. In fact, several cell lines have been reported to undergo *in vitro *transformation into a malignant phenotype [27-29]. Conversely, human tumours implanted *s.c. *in nude mice are known to have little metastatic capability [30]. However, KGN cells were found to be metastatic with nodule formation occurring mostly in the bowel. Because the nodules were formed in the submucosa of the small intestine, they must have not metastasized peritoneally. However, the route of KGN cells to the bowel remains uncertain. Although it seems that the metastases develop slowly since the tumour nodules of KGN cells remained small in size after 3 months of transplantation and histological evaluation (3 mm × 3 mm), this cell line may be useful for research on metastasis.

## Conclusion

We characterised KGN cells as a malignant tumour model of GCTs. Continuously cultivated KGN cells acquire an aggressive phenotype, confirmed by the analysis of cellular activities and the expression of biomarkers. More strikingly, KGN cells injected under the skin were metastatic with nodule formation occurring mostly in the bowel. Thus, this cell line is a good model for analysing GCT progression and the mechanisms of metastasis.

## Competing interests

The authors declare that they have no competing interests.

## Authors' contributions

MI conducted cell culture, the analysis of cellular behaviour and prepared the manuscript. MM performed Western blot analysis. KT designed the experiments on the histological analysis. HS performed in the histological evaluation. MS participated in the design of study. YT coordinated the study and finalised the preparation of the manuscript. All authors have read and approved the final manuscript.

## Authors' information

MI's current address: Cancer Cell Circuitry Laboratory, Institute of Biomedicine/Biochemistry and Genome-Scale Biology Program, Biomedicum Helsinki, University of Helsinki, Helsinki, Finland.

MM's current address: Department of Biomedical Science, Graduate School of Agricultural and Life Sciences, The University of Tokyo, Tokyo, Japan.

## Pre-publication history

The pre-publication history for this paper can be accessed here:


